# Interference with AGEs formation and AGEs-induced vascular injury mediates curcumin vascular protection in metabolic syndrome

**DOI:** 10.1038/s41598-019-57268-z

**Published:** 2020-01-15

**Authors:** Osama A. A. Ahmed, Hany M. El-Bassossy, Ahmad S. Azhar, Mayada M. Tarkhan, Mahmoud M. El-Mas

**Affiliations:** 1 Department of Pharmaceutics, Faculty of Pharmacy, King Abdulaziz University, Jeddah, KSA Saudi Arabia; 2 Department of Pharmaceutics and Industrial Pharmacy, Faculty of Pharmacy, Minia University, Minia, Egypt; 3 Department of Pharmacology and Toxicology, Faculty of Pharmacy, Zagazig University, Zagazig, Egypt; 4 Pediatric Cardiac Center of Excellence, Faculty of Medicine, King Abdulaziz University, Jeddah, KSA Saudi Arabia; 5 Department of Biochemistry, Faculty of Science, King Abdulaziz University, Jeddah, KSA Saudi Arabia; 6 Department of Pharmacology and Toxicology, Faculty of Pharmacy, Alexandria University, Alexandria, Egypt

**Keywords:** Pharmacology, Metabolic syndrome

## Abstract

Vascular dysfunction predisposes to cardiovascular complications of metabolic syndrome (MetS). The current study investigated the mechanism(s) of curcumin’s (CUR) protective effect against vascular reactivity irregularities in MetS. MetS was induced by feeding rats on high fructose high salt diet. Tension studies were undertaken in aortic rings to assess the influence of CUR on vasoconstrictor or vasorelaxant responses. The effect on advanced glycation endproducts (AGEs) was studied by incubating aortic tissues with methylglyoxal, the AGEs precursor, in the absence and presence of CUR. In addition, CUR effects on *in-vitro* generation of AGEs and diphenyl-2-picrylhydrazyl (DPPH) free radicals were studied. The incubation with CUR for 1 hr produced significant and concentration-dependent alleviation of the exaggerated vasoconstriction observed in aortas isolated from MetS, however failed to improve the concomitant attenuation of vasodilatory responses to ACh in PE-precontracted aortas. By contrast, CUR caused direct concentration-dependent vasodilations of precontracted aortas, effects that were blunted after nitric oxide synthase inhibition by L-NAME. Similar to its effects in MetS aortas, CUR alleviated exaggerated PE vasoconstriction but did not affect impaired ACh vasodilations in AGEs-exposed aortas. In addition, CUR showed significant dose-dependent DPPH free radicals scavenging activity and inhibited both MG and fructose induced AGEs formation at the level of protein oxidation step as evident from the effect on dityrosine and N-formylkyramine. CUR alleviates exaggerated vasoconstriction in MetS through interfering with AGEs formation and AGEs-induced vascular injury. Free radical scavenging and direct vasodilatory activities could also participate in the advantageous vascular actions of CUR.

## Introduction

Metabolic syndrome (MetS), also referred to as insulin resistance syndrome or syndrome X, is characterized by a cluster of conditions, namely: central obesity, dyslipidemia, hypertension and hyperglycemia^1^. The detrimental effect of MetS in general is its close association with the development of cardiovascular diseases (CVD) and type 2 diabetes mellitus. A major indicator of the cardiovascular complications associated with MetS is vascular damage which is translated in to exaggerated contraction and attenuated dilation of the blood vessels in response to vasoconstrictors and vasodilators respectively^2^. Hypertension leading to arterial wall stiffness, as well as atherosclerosis secondary to dyslipidemia, are some of the MetS conditions which catalyze the appearance of the vascular dysfunction observed^3,4^. Another MetS related reaction negatively impacting vascular function is glycation, which occurs between proteins and monosaccharide sugars, typically elevated in MetS^5^. Methylglyoxal (MG), a highly reactive sugar derivative, is also greatly elevated during hyperglycemia and is capable of reacting with proteins^5^. The resultant glycation products are known as advanced glycation end products (AGEs) and are accountable for enhanced oxidative stress and inflammatory levels as well as lowered nitric oxide (NO) vasodilator levels, all which contribute to vascular damage^6^. Furthermore the glycation process is associated with protein oxidation reactions leading to the formation of glycoxidation products namely dityrosine and N‘-formylkynurenine from the oxidation of tyrosine and tryptophan amino acids residues respectively^7^. These products may then be measured as indicators of the extent of oxidative protein damage^8^.

Given the detrimental effects MetS has on health, there is a growing interest in the use of natural products, specifically plant polyphenols, combined with the present synthetic drugs for better control of the disease^9^. One of such compounds is curcumin (CUR), a naturally found compound obtained from turmeric, a member of the ginger family^10^. Belonging to the curcuminoid group, CUR has a polyphenolic structure and as with most polyphenols, imparts a distinct color to the plant from which it originates; in this case bright yellow^11^. Various potential therapeutic uses of CUR have already been studied such as in cancer, gastrointestinal and cardiovascular diseases, Alzheimer’s and more recently, its anti-depressant effect^12^.

CUR showed favorable effects on MetS components, such as dyslipidemia^13^, hypertension^14^ and insulin resistance^15^. Published work from our laboratory showed beneficial vascular effects of CUR under different pathological conditions; CUR protected from inflammation-induced exaggerated pulmonary artery vasoconstriction^16^. CUR also alleviated exaggerated vasoconstriction of aortas incubated with high fructose^17^. Chronic administration of CUR protected from the hypertension and vascular dysfunction associated with diabetes^18^ and metabolic syndrome^19^. However, direct vascular effects of CUR and the exact mechanism of action still remain unclear.

As a follow up to our previous reports, the current study tested the hypothesis that inhibition of AGEs accumulation and dampening down of concomitant oxidative damage underlie to the vasculoprotective action of CUR in rats with MetS. More support for such postulate was sought by assessing the effect of CUR on vascular and oxidative anomalies induced by methylglyoxal (MG).

## Materials and Methods

### Drugs and chemicals

Bovine serum albumin (BSA), aminoguanidine (AG), methylglyoxal (MG), fructose, acetylcholine (ACh), phenylephrine (PE), Nω-Nitro-L-argininemethyl ester hydrochloride (L-NAME), diphenyl-2-picrylhydrazyl (DPPH) as well as the natural compound CUR were all purchased from Sigma–Aldrich, Dorset, UK. Ultrapure deionized water was used as solvent except for the natural compounds and DPPH which were dissolved in dimethyl sulfoxide (DMSO) in a concentration which did not exceed 0.1% in the reaction media. All other solvents and chemicals used were of analytical grade.

### Experimental animals

Rats used in this study were male Wistar 6–8 weeks old rats weighing 180–200 g (King Fahd Medical Research Center, King Abdulaziz University, Jeddah, Saudi Arabia). Clear polypropylene cages were used to house 4 rats each and included access to purified water and standard rodent pellets. Constant animal housing conditions applied constituting of alternating 12 hours light and dark, a temperature of 22 ± 3 °C, a relative humidity of 50–60% and adequate ventilation. Experimental protocol was approved by the Research Ethical Committee, Faculty of Pharmacy, King Abdulaziz University, Jeddah, Saudi Arabia (approval number 1071439) and was conducted in accordance with the Saudi Arabia Research Bioethics and Regulations.

MetS was induced in rats by adding fructose (10%) to drinking water and NaCl (3%) to food pellets. This regimen began in 6-week- old rats and continued for 12 weeks. Fructose was dissolved completely in drinking water while NaCl was dissolved in a small volume of water, sprayed on food pellets and left to air dry. Control rats received regular food pelletst. The development of MetS was verified via the assessment of systolic blood pressure and fasting serum levels of insulin, glucose, fructosamine, cholesterol and triglycerides, as described in our previous studies^20^. Histopathological examination of paraffin-embeded thoracic aorta cross sections was carried out using haematoxylin and eosin stain as previously reported^21^.

### Effect of CUR on MetS- induced exaggerated vasoconstriction and impaired dilatation

Isolated thoracic aorta obtained from MetS rats was used firstly to demonstrate the vascular damage associated with MetS and secondly to investigate the ability of CUR to remedy such damage by ameliorating the vasoconstriction and improving the vasodilation. The isolated artery technique described by El-Bassossy *et al*.^22^ was used to study the vascular reactivity. Aorta obtained from control rats was cleaned off fats and cut in to 3 mm rings. Each ring was suspended in automated organ bath (Panlab, Barcelona, Spain) channels filled with 25 ml of Krebs Henseleit buffer (118 mM NaCl, 4.8 mM KCl, 2.5 mM CaCl_2_, 1.2 mM MgSO_4_, 1.2 mM KH_2_PO_4_, 25 mM NaHCO_3_ and 11.1 mM glucose). The buffer medium, changed every 30 minutes, was continuously aerated with 95% oxygen and 5% carbon dioxide gas and maintained at 37 °C. Aortic tension was quantified by an isometric force transducer (ADInstruments, Bella Vista, Australia) and the result displayed through the PowerLab Data Interface Module connected to a PC running Chart software v8 (ADI Instruments).

Aortic segments were directly incubated with CUR (10 and 30 µM) or its vehicle (0.1% DMSO) for 1 hr. Afterwards, the aortic vaso-reactivity was studied by cumulative additions of PE concentrations (10^−8^ to 10^−5^ M) followed by cumulative additions of ACh (10^−8^ to 10^−5^ M). Contractions were presented in mg tension while relaxations were calculated as percentages of the PE-induced contraction. Aortae from the same animals were divided between different experiments so that each group experiments were performed on aortae isolated from 8 animals.

### Effect of CUR on AGEs induced exaggerated vasoconstriction and impaired dilatation

MG was used to induce vascular damage to isolated thoracic aorta obtained from naive rats and CUR was investigated for its ability to overcome the resultant exaggerated vasoconstriction and impaired dilatation. The above described isolated artery techniques were applied with some differences. The aortic rings were left for 20 minutes to accommodate to the surrounding conditions and reach equilibrium at a 1500 mg ± 50 resting tension. Primary contraction and relaxation of the aorta was then performed by adding PE (10^−5^ M) and ACh (10^−5^ M) respectively. After the tension was returned to resting state, MG (100 µM) was added in the absence and presence of CUR (10–100 µM) to aortic segments and left for incubation at 37 °C for 1 h. For control preparations, 0.1% DMSO solution was added instead of MG. Following the incubation period, cumulative concentrations of PE and ACh (10^−8^ to 10^−5^ M each) were added as previously described.

### Effect of CUR on AGE formation

The effect of curcumin on AGE formation was studied as previously reported by Abdallah *et al*.^23^. In a black 96- well plate, 180 μl of bovine serum albumin (BSA,10 mg/ml in PBS) containing curcumin (10–30 µM) or the standard anti-glycation compound, aminoguanidine (AG, 1 mM), were added in the wells. To these mixtures, 20 μl of AGEs precursor, methylglyoxal (MG 50 mM) freshly prepared was added while 20 μl of PBS were added for the blank. Control row of wells in which PBS was added instead of MG to BSA containing CUR was prepared in order to test any autofluorescence at the used wavelengths (no significant CUR autofluorescence was detected). The plate was left for incubation at 37 °C for one hour in the dark. Fluorescence intensity of the AGE produced was measured at λex = 325 and λem = 440 nm with the use of Monochromator SpectraMax® M3 plate reader (Molecular Devices, Sunnyvale, CA, USA). Dityrosine and N′-formylkynurenine levels were quantified through fluorescence intensity measurements at λex = 330 and 325 nm; λem = 415 and 434 nm^24^. Experiments have independently been repeated three times for each group.

The experiment was repeated as above but instead of MG, fructose (which has been used to induce MetS fructose) was used at concentration of 50 mM. The reaction containing fructose (50 mM) and CUR (10–30 µM) was incubated at 37 °C for two weeks in dark eppendorf tubes rather than in the plate to avoid evaporation or contamination of the reaction mixture. The relatively long incubation time pressed for the need of sodium azide (0.02%) added to inhibit bacterial growth. The samples were then transferred to the plate, after the two-week incubation period, for reading. Fluorescence intensities were measured as before. The two weeks have shown to be sufficient time for interaction between sugars and BSA to form measurable AGEs in previous studies^25^.

### Free radical scavangering effect of CUR

The reactive oxygen species scavenging ability of CUR was studied as previously reported with some modifications^26^. In a clear 96- well plate, CUR (10–30 µM) in methanol was added to DPPH solution (240 µM) in methanol/tris (1:1 v/v). For the blank, methanol was used instead of CUR. DPPH was prepared immediately before addition to the plate. The vehicle, methanol/tris (1:1 v/v) was added instead of DPPH to a control row of wells containing the same concentrations of CUR in order to exclude any auto absorbance (none was significant). The absorbance was measured at 520 nm every minute for 10 minutes with the use of Monochromator SpectraMax® M3 plate reader (Molecular Devices, Sunnyvale, CA, USA). Three independent experiments have been performed for each group.

### Direct vasodilation of CUR

This was conducted to assess whether curcumin possesses a direct relaxant effect on the aortic smooth muscles and whether this effect is through stimulating the release of the vasodilator NO from the vascular endothelium. Isolated artery techniques described above were applied. The MetS aortic segments were directly incubated with or without L-NAME (1 mM) at 37 °C for 30 minutes. After incubation period, vasoconstriction was achieved through the addition of PE 10^−5^ M. Once the contraction reached a plateau, CUR was added in an ascending order of concentrations (10–100 µM), giving time for the relaxation to reach plateau before each addition. DMSO (0.1%) was used as a control. After the three CUR concentrations, ACh 10^−5^ M was added to achieve complete relaxation.

### Statistical analysis

Values are expressed as mean ± standard error of the mean. Vascular reactivity, vasodilation and DPPH data compared by two-way analysis of variance (ANOVA) followed by Newman-Keuls’ post hoc test. AGEs formation data was compared by one way ANOVA followed by Newman-Keuls’ post hoc test while MetS parameters were analyzed by unpaired student t test using Prism 5® software (Graphpad, CA, USA). Statistical significance was considered when P value < 0.05. The sample size was calculated using G*Power 3.1.9.4 free software (Kiel University, Germany) at statistical power value of 0.8 and α value of 0.05. This has been clarified in the methods section.

## Results

### Effect of high fructose/high salt diet feeding

**Figure 1:**
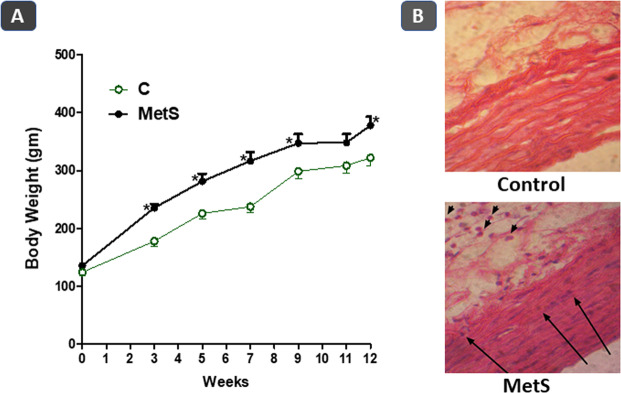
Effect of feeding rats on high fructose (10% in drinking water), high salt (3%) diet for 12 weeks on weekly body weight (**A**) and thoracic aorta histopathology. (**B**) Results are expressed as mean ± SEM (n = 8 for all groups). *p < 0.05 when compared to the corresponding control values, *p < 0.05 when compared to the corresponding MetS values using unpaired student t test. Arrows point to leukocyte infiltration and endothelial pyknosis.

Feeding rats on high fructose (10% in drinking water) and high salt (3%) diet led to significant increase in body weight gain after 3, 5, 7, 9 and 12 weeks compared with control rats (p < 0.05, Fig. 1A). The high fructose/high salt group consumed 3.731 ± 0.5272 g fructose/day. In addition, the twelve-week administration of the high fat/high salt diet lead to marked structural changes; leukocytes infiltration in the adventitia and cellular pyknosis in thoracic aorta isolated from those rats. Furthermore, high fructose/high salt diet feeding led to significant elevations in systolic blood pressure and serum levels of insulin, glucose, fructosamine, total cholesterol and triglycerides as shown in Table 1.

**Table 1:** Effect of feeding rats on high fructose (10% in drinking water), high salt (3%) diet for 12 weeks on systolic blood pressure (SBP) fasting serum insulin, glucose, fructosamine, total cholesterol (TC) and triglycerides (TG).

	SBP (mmHg)	Insulin (ng/ml)	glucose (mg/dl)	Fructosamine (μmol/l)	TC (mg/dl)	TG (mg/dl)
Control	100.6 ± 1.72	2.6 ± 0.38	77.00 ± 2.28	47.47 ± 2.25	89.0 ± 3.54	44.5 ± 6.06
MetS	122.7 ± 2.84*	6.6 ± 0.91*	106.20 ± 3.46*	74.88 ± 5.32*	123.2 ± 11.89*	88.5 ± 14.29*

Data is presented as mean ± standard error of the mean for n = 5–8. *Significantly different from the control values at p < 0.05 using unpaired student t test.

### Effect of CUR on MetS-induced exaggerated vasoconstriction and impaired dilatation

Figure (1A) shows that there was exaggerated vascular contraction seen with MetS aortas in response to PE when compared to control tissues. This is indicated by the significantly higher contractions seen in MetS aortas when exposed to the two higher concentrations of PE. Incubation of MetS aortas with CUR 30 µM at 37 °C for 1 h significantly reduced the exaggerated contraction back to control levels while CUR 10 µM has no apparent effect on the vasoconstriction.

Concerning the vascular relaxation, Fig. (1B) shows that the MetS aorta experienced impaired smooth muscle dilatation in response to ACh when compared to control. This observed impaired dilation was not amended by any of the CUR concentrations applied.

### Effect of CUR on MG induced exaggerated vasoconstriction and impaired dilatation

**Figure 2:**
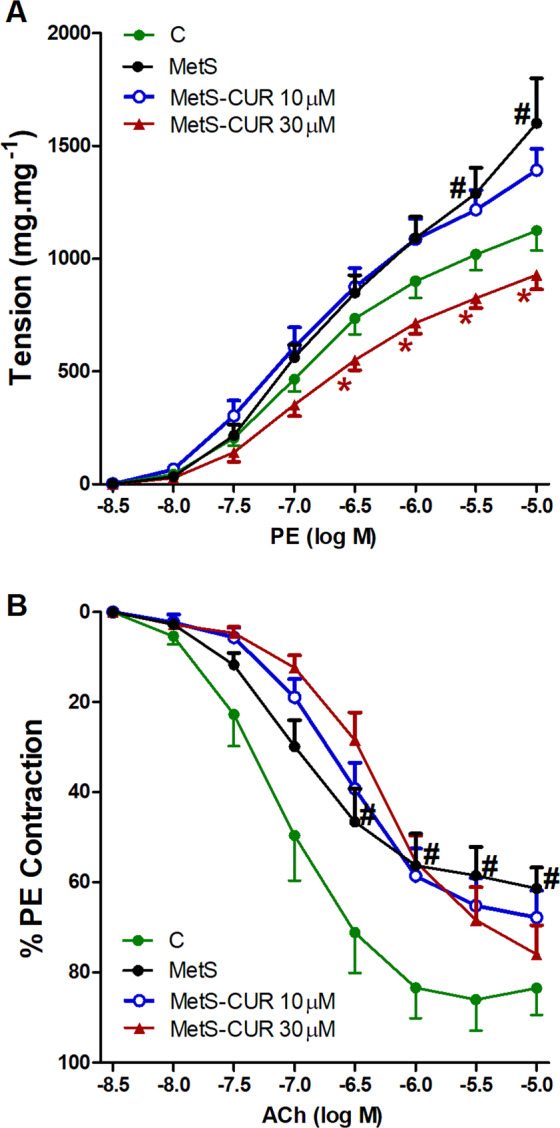
Effect of curcumin (CUR, 10 or 30 μM) one hour incubation on responses to phenylephrine (**A**) and Ach (**B**) of aortas isolated from metabolic syndrome (MetS) animals. Results are expressed as mean ± SEM (n = 8 for all groups except for the control group in case of Ach n = 7). ^#^p < 0.05 when compared to the corresponding control values, *p < 0.05 when compared to the corresponding MetS values by two-way ANOVA followed by Bonferroni post-hoc test.

As shown in the Fig. (2A), the pre-incubation of aortic rings with MG caused significant increases in cumulative contractions induced by subsequent exposures to PE when compared to control (non-MG treated) aortas. The high CUR concentration (30 µM) significantly reduced the exaggerated contraction back to control levels while CUR 10 µM had no observed effect.

Similar to the effect seen in MetS, the exposure of aortic rings to MG (100 µM) caused downward shifts in the concentration-vasorelaxant response curves of ACh and significant decreases in ACh relaxations compared with the relaxant responses demonstrated in control preparations. The co-incubation of aortic rings with none of CUR concentrations (10–30 µM) could reverse the depressant effect of MG on ACh responses (Fig. 2B).

### Effect of CUR on AGE formation

**Figure 3:**
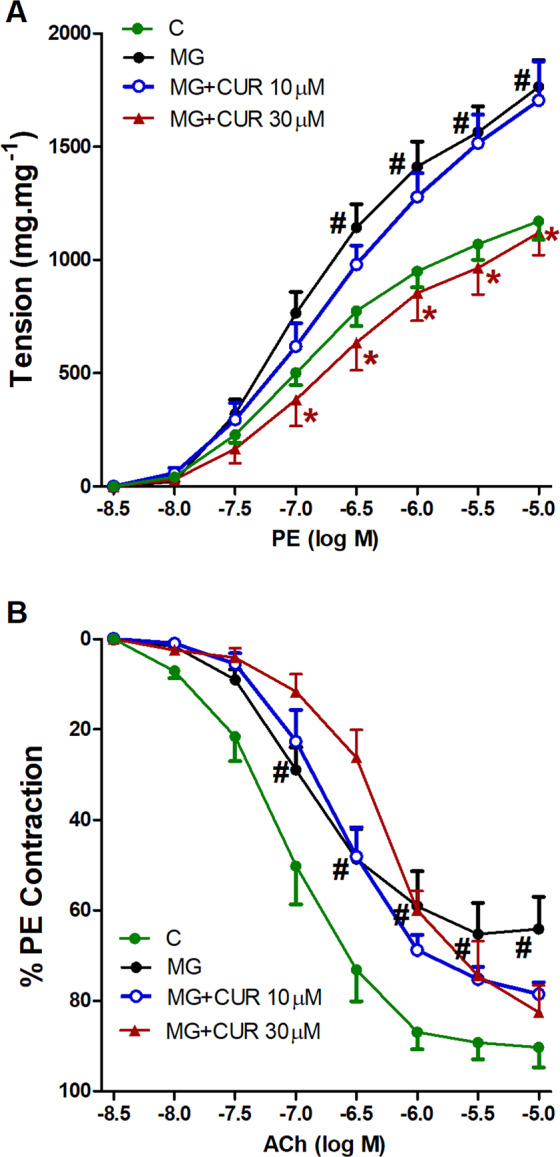
Effect of curcumin (CUR, 10 or 30 μM) one hour incubation on responses to phenylephrine (**A**) and Ach (**B**) of aortas exposed to advanced glycation end-products (AGEs) injury using the AGEs precursor, methylglyoxal (MG). Results are expressed as mean ± SEM (n = 8 for all groups except for MG + CUR 10 μ in phenylephrine and MG + CUR 30 μ Ach n = 7). ^#^p < 0.05 when compared to the corresponding control values, *p < 0.05 when compared to the corresponding MG values by two-way ANOVA followed by Bonferroni post-hoc test.

Figure 3A shows that incubation of BSA (10 mg/ml) with MG (50 mM) at 37 °C for one hour resulted in a significant increase in AGE production compared with the control. The addition of AG (1 mM) to the reaction mixture significantly suppressed the AGE formation to levels similar to that of control values. CUR (10–30 µM) caused significant and concentration-dependent inhibition of MG-mediated AGEs formation. The effect is shown to have been most prominent at the high CUR concentration added (30 µM).

The protein oxidation products dityrosine and N-formyl kynurenine, (Fig. 3B,C) were affected by the intervening drugs in a pattern similar to that of AGEs. Both oxidation products were significantly elevated upon incubation of MG (50 mM) with BSA (10 mg/ml), and their levels were significantly reduced in reaction mixtures containing AG (1 mM) or CUR (10–30 µM).

**Figure 4:**
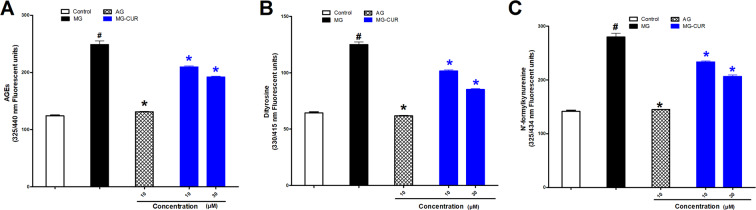
Effect of curcumin (CUR, 10 or 30 μM) one hour incubation on advanced glycation end-products (AGEs) (**A**) and the glycosylation products dityrosine. (**B**) N-formylkyranine (**C**) using the AGEs precursor, methylglyoxal (MG). Results are expressed as mean ± SEM (n = 3 independent experiments). ^#^p < 0.05 when compared to control, *p < 0.05 when compared to MG by one-way ANOVA followed by Bonferroni post-hoc test.

In terms of the fructose driven glycation reaction, Fig. 4 A demonstrates that the BSA incubation with fructose at 37 °C for two weeks resulted in significant AGE and protein oxidation products formation compared to control values. Similarly, the production of these constituents was suppressed by AG (1 mM). Both CUR concentrations (10 and 30 µM) significantly inhibited the AGEs formation driven by fructose. As for the protein oxidation products (Fig. 4B,C), both were reduced by CUR (10–30 µM).

### Anti-oxidant effect of CUR

**Figure 5:**
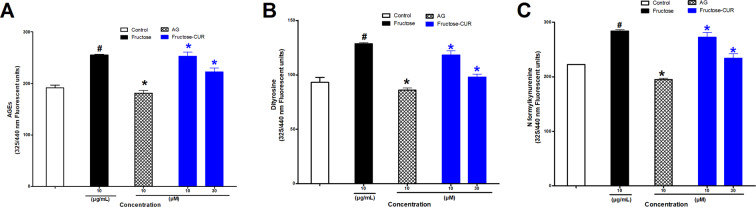
Effect of curcumin (CUR, 10 or 30 μM) incubation on advanced glycation end-products (AGEs) (**A**) and the glycosylation products dityrosine (**B**), N-formylkyranine (**C**) using fructose. Results are expressed as mean ± SEM (n = 3 independent experiments). ^#^p < 0.05 when compared to control, *p < 0.05 when compared to fructose by one-way ANOVA followed by Bonferroni post-hoc test.

According to the 10 minutes reaction between DPPH (240 µM) and CUR (10–30 µM) shown in Fig. 5, it is clear that both CUR concentrations (10 and 30 µM) possess free radical scavenging activity. CUR 30 µM is shown to have a more significant effect, scavenging almost 40% of the present DPPH free radicals after 10 minutes.

### Direct vasodilation of CUR

**Figure 6:**
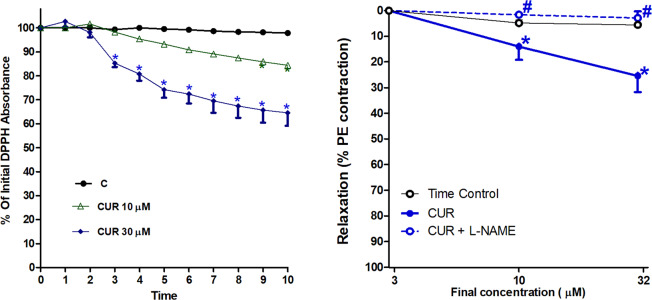
Effect of curcumin (CUR, 10 or 30 μM) incubation on DPPH free radical (**A**), Effect of curcumin (CUR) cumulative addition with or without L-NAME (1 mM) on phenylephrine (PE, 1μ)-precontracted isolated aortas. (**B**) Results of DPPH are expressed as mean ± SEM (n = 3 independent experiments at each time point). *p < 0.05 when compared to the corresponding time control values. Vasodilation results are expressed as mean ± SEM (n = 7 for all groups except for CUR group n = 6). *p < 0.05 when compared to time control values, ^#^p < 0.05 when compared to the corresponding CUR values by two-way ANOVA followed by Bonferroni post-hoc test.

According to the results shown by Fig. 6, direct addition of cumulative concentrations of CUR to PE (10^−5^ M)-precontracted aortas brought about a decrease in tension and hence concentration-dependent vasodilations. The aortic relaxation effect was significant starting from 10 µM concentration, whereby approximately 30% relaxation of the initial contraction was achieved by CUR 30 µM. The aorta incubated with L-NAME (1 mM) at 37 °C for 30 minutes showed no response to any of the CUR concentrations used and thus the relaxation effect was completely inhibited.

## Discussion

This is the first study to our knowledge which investigates the direct *in- vitro* vasculo- protective effects of CUR on isolated thoracic aorta obtained from MetS animal. Results show that CUR significantly alleviated the exaggerated vasoconstriction induced by MetS or by *in-vitro* aortic exposure to MG, however failed to improve the associated reductions in cholinergically-mediated vasodilations. The inhibition of AGEs formation and perhaps AGEs-induced vascular damage contribute to CUR vasculoprotection. Furthermore, the antioxidant effect of CUR could participate in its effect on AGEs and thus protect from the detrimental effects which AGEs can inflict on vasculature.

Previous studies have investigated the vascular damage associated with MetS, which encompasses impairment of vascular reactivity^27^ and histopathological alterations in the vascular architecture^28,29^. In the current study, aortas excised from MetS animals exhibited exaggerated vasoconstriction together with impaired vasodilation responses. *In vitro* incubation with CUR completely prevented such exaggerated vasoconstriction. This result is in accordance with findings of Majithiya and Balaraman^30^ who demonstrated CUR’s alleviation of the exaggerated vascular contraction present in diabetes, especially in early stages of the disease.

Different approaches have been implemented in search for the possible mechanism(s) responsible for CUR alleviating the exaggerated vasoconstriction associated with MetS. The first was studying CUR’s effect on endogenous vasodilation. Improving impaired vasodilation is an indirect way of alleviating vasoconstriction by many compounds as reported previously^31^. In the current study, the same dose regimen of CUR failed to improve the impaired vasodilation associated with MetS, thereby inferring other mechanisms could be involved in the compound’s alleviation of the intensified vascular contraction.

Therefore, we tested the hypothesis that the protection form AGEs underlies the rectifying action of CUR on vascular damage induced by MetS. In a very similar fashion to that found in MetS aortas, we found that CUR alleviates the exaggerated vasoconstriction induced by direct AGEs exposure. Given the very important role of AGEs in the vascular dysfunction in MetS^5^, this point to a key role for the anti-glycation in mediating CUR vasculo-protective effect.

CUR’s effect on AGEs formation was investigated as well using the AGEs precursor MG. The use of MG in the glycation reaction with BSA has been applied previously considering that MG is the most reactive precursor for AGE formation and thus ensures a fast reaction^24^. Similarly, fructose was chosen as the reducing sugar to investigate glycation rather than glucose, because it has been used to induce MetS in the current study in addition to the much faster reaction between fructose and protein^32^. In the current study, CUR prevented in a dose-related fashion AGE production induced by MG or fructose. A recent study conducted by Sun *et al*.^33^ reported inhibition of AGE induced oxidative stress by CUR in endothelial cell.

Results of the current study also suggest that CUR could execute its anti-glycation effects through reducing the oxidative insults indicated by CUR’s ability to scavenge DPPH free radicals. AGE and oxidative stress are strongly correlated given that the glycation reaction involves protein oxidation through the tendency of the generated AGEs to directly facilitate the production of reactive oxygen species through interaction with the AGE receptor RAGE^6^. In the current study, CUR not only inhibits the formation of the final AGEs products but also inhibits the protein oxidation products dityrosine and N-formyl kynurenine at the same concentration levels. This points to a very important role of the antioxidant effect of CUR in its anti-glycation activities. The clear anti-oxidant activities of CUR at the same concentrations shown by DPPH assay in the current study, support this assumption further. The DPPH assay employed in this present study has been previously utilized to determine the anti-oxidant activity of numerous naturally occurring polyphenolic compounds^34–36^.

Other than the anti-glycation and anti-oxidant effect, CUR may also protect the vascular structure from MetS inflicted injury by NO related mechanisms. Such premise receives support from the observation that CUR caused direct relaxation of pre-contracted aorta. Considering that the direct relaxant effect of CUR completely disappeared after NOS inhibition by L-NAME, it is plausible that the activation of endothelial nitric oxide synthase (eNOS) might account for the CUR-evoked vasorelaxation. The facilitating action of CUR on NO-mediated vasorelaxations has been attributed to the enhancement of endothelial function by increasing NO bioavailability^37^ and eNOS expression^14^. CUR also acts indirectly to maintain NO levels by reducing AGEs, which are known to quench NO^6^.

Of note, the presumed NO-dependent relaxant effect of CUR does not appear to reconcile with our finding that the MetS- or MG-evoked attenuation of ACh relaxation was preserved in CUR-treated preparations. This may be explained by the findings of Fang *et al*.^38^, who showed that prior, but not consequent, CUR treatment effectively reverses vasodilation dysfunction provoked by *in-vitro* incubation of aortas with high glucose levels. It is possible, therefore, that a favorable effect for CUR could be only seen when a prophylactic regimen is assumed. It should also be considered that the vasodilatory response elicited by ACh is multifactorial, involving both NO-dependent and independent cellular pathways^39,40^. A differential interaction of CUR with these cellular events might possibly explain its little or no capacity in combating the reduced Ach vasodilations in MetS aortas. Additionally, evidence obtained from our laboratory^41^ and others^42^ links NOS-derived NO to vasodilation caused by the activation of cholinergic sites as well as by several other vasodilator stimuli. These multiple cellular events have been shown to be distinctly affected by vasculotoxic insults and corrective measures^40,43,44^. More studies are apparently needed to precisely identify the cellular mechanism(s) of the NO-dependent vasodilatory action of CUR.

It is important to comments on possible strengths and limitations of the current research. The investigation of the multi-factorial processes involved in provoking MetS vasculotoxicity is a clear advantage of this investigation. Moreover, the study highlights the therapeutic potential of CUR is mitigating pathophysiologic consequences that trigger vascular anomalies of MetS. That said, it should be noted that further research is required to assess the determine whether the beneficial effects of CUR could be replicated when the drug is administered to whole animal and human preparations of MetS.

## Conclusion

The study shows that CUR possesses a vasculo-protective effect in MetS through its inhibitory influences on AGEs accumulation and associated oxidative insult. Clinically, the data highlight the therapeutic potential of CUR as adjunct therapy for MetS management.
