# Correction: Interference with AGEs formation and AGEs-induced vascular injury mediates curcumin vascular protection in metabolic syndrome

**DOI:** 10.1038/s41598-026-57991-4

**Published:** 2026-06-25

**Authors:** Osama A. A. Ahmed, Hany M. El-Bassossy, Ahmad S. Azhar, Mayada M. Tarkhan, Mahmoud M. El-Mas

**Affiliations:** 1 Department of Pharmaceutics, Faculty of Pharmacy, King Abdulaziz University, Jeddah, KSA Saudi Arabia; 2 Department of Pharmaceutics and Industrial Pharmacy, Faculty of Pharmacy, Minia University, Minia, Egypt; 3 Department of Pharmacology and Toxicology, Faculty of Pharmacy, Zagazig University, Zagazig, Egypt; 4 Pediatric Cardiac Center of Excellence, Faculty of Medicine, King Abdulaziz University, Jeddah, KSA Saudi Arabia; 5 Department of Biochemistry, Faculty of Science, King Abdulaziz University, Jeddah, KSA Saudi Arabia; 6 Department of Pharmacology and Toxicology, Faculty of Pharmacy, Alexandria University, Alexandria, Egypt

Correction to: *Scientific Reports* 10.1038/s41598-019-57268-z, published online 15 January 2020

This Article contains errors.

The Authors performed experiments which were conducted simultaneously under identical experimental conditions, including the same animal batches, treatment protocols, laboratory settings, and analytical procedures. The negative and positive control data (“C” and “MG”) shown in Figures 3A, 3B, and 6A were previously published by the authors^1^ and were reused in the present study for comparative and interpretative purposes. This reuse was undertaken to ensure consistency between studies and to avoid the unnecessary use of additional experimental animals, in line with internationally accepted ethical standards and the 3Rs principles of animal research (Replacement, Reduction, and Refinement).
